# Changes in identity and habit formation during 3 months of sport and physical activity participation among parents with young children

**DOI:** 10.1111/aphw.70009

**Published:** 2025-02-11

**Authors:** Ryan E. Rhodes, Colin M. Wierts, Mark R. Beauchamp, Valerie Carson, Sandy Courtnall, Chris M. Blanchard

**Affiliations:** ^1^ School of Exercise Science, Physical and Health Education University of Victoria Victoria Canada; ^2^ School of Kinesiology Louisiana State University Baton Rouge LA USA; ^3^ School of Kinesiology University of British Columbia Vancouver Canada; ^4^ Faculty of Kinesiology, Sport, and Recreation University of Alberta Edmonton Canada; ^5^ Faculty of Medicine Dalhousie University Halifax Canada

**Keywords:** behavioral maintenance, exercise, parenthood, recreational sport

## Abstract

Understanding factors that might contribute to sustained moderate‐to‐vigorous intensity physical activity (MVPA) after the initial start of participation is important. The purpose of this study was to examine the changes in PA habit and identity, two constructs purported to drive behavioral maintenance, among parents of young (< 13 years of age) children participating in a PA intervention across 3 months. Parents (team sport *n* = 58; individual PA *n* = 60; control *n* = 66) completed measures of PA habit, PA identity, and self‐reported MVPA at baseline, and post‐randomization at 6 weeks and 3 months. Identity and habit showed an increase across time, and these changes interacted with group condition assignment. Identity increased for participants in the team sport condition and was significantly different from a stable profile among those assigned to the control condition. Participants in the individual PA condition increased identity from 6 weeks to 3 months. Habit had a more attenuated change across time, with participants in the team sport condition showing an increase from 6 weeks to 3 months compared with the other conditions. The findings contribute to our understanding of maintenance processes and represent the first exploration of how involvement in team sport might shape subsequent habit and identity development.

## INTRODUCTION

Moderate‐to‐vigorous intensity physical activity (MVPA) is associated with improvements in several short‐term health indicators (e.g., blood pressure, insulin sensitivity, sleep, anxiety, and cognition) as well as longer term benefits such as preventing obesity, reducing the risk of several cancers, improved mental health, and limiting the progression of current chronic diseases or conditions (US Department of Health and Human Services, 2018; Ross et al., 2020). Recreational sport participation, as a form of physical activity among adults, is associated with several additional important psychosocial outcomes (e.g., psychological well‐being/life satisfaction, lower stress, higher social functioning, greater vitality, and a sense of community belonging) when compared with adults who do not participate in sports (Blake et al., 2022; Eime et al., 2013; Gayman et al., 2016; Kim et al., 2020).

Despite these substantive mental and physical health contributions, one third of adults do not meet international aerobic MVPA recommendations of 150 min or more per week (Strain et al., 2024), with physical inactivity even higher among high‐income nations. For example, over 40 per cent of Canadian adults are insufficiently physically active with respect to those guidelines (Strain et al., 2024). When considering sport participation specifically, participation rates are even lower. In Canada, sport participation declines to 23 per cent in adulthood from over half of Canadians participating in sport during late adolescence (Canadian Heritage, 2012; Statistics Canada, 2023). Even among the regions of the world where sport participation is most prevalent (e.g., Australia, Scandinavia, Northern Europe), roughly two‐thirds of adults report that they do not engage in regular recreational sport (Eime et al., 2015; European Commission, 2022). Furthermore, some adult groups may show lower sport and PA participation rates than others, underscoring the importance of underserved population promotion efforts (Ding et al., 2024). One such group is parents of young children, who report significant declines in overall MVPA, and this includes sport participation (Abbasi & Akker, 2015; Bellows‐Riecken & Rhodes, 2008; Carson et al., 2018). Thus, a focus on sustaining MVPA during early parenthood is warranted.

Understanding the factors driving adult sport and MVPA participation is critical to promotion initiatives. In particular, an increasing focus for health promoters is to understand factors that may be instrumental in maintaining the behavior after the initial start of participation (Dunton et al., 2022; Rhodes & Sui, 2021). This is also highly relevant among parents of young children, because MVPA declines during early parenthood are typically from a failure to maintain a prior active lifestyle due to the demands of parenthood (Bellows‐Riecken & Rhodes, 2008; Candelaria et al., 2012). Thus, any new initiative to promote MVPA and sport will likely be faced with those same demands to maintain MVPA. Many factors, which are socioecological in nature, may be key in understanding ongoing MVPA participation (see Kwasnicka et al., 2016 for a review). Two psychological factors that are often purported to be linked to maintaining health behaviors like MVPA are habit and identity. Specifically, habit and identity are considered central to behavioral maintenance because behavioral performance experiences are a pre‐requisite to their formation (Burke, 2006; Wood & Runger, 2016), and formation is dynamic and develops over time from behavioral initiation (Spruijt‐Metz et al., 2015). Further, habit and identity purportedly influence behavior reflexively (Burke & Stets, 2022), thereby reducing the requirement for effortful self‐regulation (Caldwell et al., 2018; Hagger, 2018; Rhodes & Sui, 2021; Wood & Neal, 2016), and both are considered resistant to change once established (Burke, 2006; Verplanken et al., 1998).

*Habit* is the process by which behavior is influenced by well‐learned cue‐behavior associations (Gardner, 2015; Rebar, 2017; Wood & Runger, 2016). Specifically, over time, as behavior is reliably performed in the same context, people can learn to associate certain cues (e.g., time of day, part of routine, locations, routine events) with the initiation of the behavior. These associations are stored in procedural memory (Gasbarri et al., 2014; Wood et al., 2022) and influence physical activity through elicitation of behavioral approach tendencies (Rhodes & Rebar, 2018). Upon experience of the cue, the approach tendency is triggered, increasing the impulse to engage in the behavior. *Identity* involves the categorization of oneself in a given role (sometimes called a role identity) and is considered a component of a multi‐dimensional, hierarchically organized, self‐concept (Burke & Stets, 2022). Identities serve as personal standards of behavior (Stryker & Burke, 2000) that allow for a dynamic, reflexive, self‐regulating control system (Burke, 2006). Specifically, the identity standard acts as a comparator to actual behavior and is activated automatically by relevant situational cues (e.g., social, environmental, performance) where the identity is either aligned or mismatched with one's behavior. Alignment experiences serve to strengthen the identity while discrepancies challenge an identity (Burke & Stets, 2022). This challenge creates negative affect and dissonance that serve to motivate identity‐consistent behavioral actions (Stets & Burke, 2000; Strachan et al., 2009).

Current research on physical activity habit and identity using observational designs has provided initial evidence for their linkage with MVPA. Three systematic reviews have aggregated the evidence of physical activity and habit (Feil et al., 2021; Gardner et al., 2011; Rebar et al., 2016), concluding that the strength of the association between habit and physical activity behavior was typically found to be moderate/strong (*r* = .43; Gardner et al., 2011; *r* = .32, Rebar et al., 2016). The basic bivariate relationship between identity and physical activity has also been established in a systematic review and meta‐analysis which found an association of *r* = .44 (Rhodes et al., 2016). There has been limited research on new parents; however, two studies by Rhodes and colleagues found that physical activity habit and identity were associated with intention‐MVPA coupling over 6 months (Rhodes et al., 2020; Rhodes et al., 2021).

While this overall research is supportive of the basic relationship between physical activity habit and identity with MVPA, only a small number of studies have focused on habit and identity development among MVPA initiates (i.e., those individuals starting and then continuing with MVPA). Research on MVPA initiates will likely best assist in the design of interventions, because these samples better reflect those who are typically the focus of promotion efforts. Kaushal and Rhodes (2015) surveyed new gym initiates for 12 weeks and found that physical activity habit formation peaked at 6 weeks but was linked to changes in MVPA across time. More recently, Baretta et al. (2024) examined the effects of cue‐behavior repetition (i.e., self‐selected cue and 15‐min walk) on physical activity habit formation across 15 weeks among low‐active individuals joining a mobile health intervention. Habit formation was idiosyncratic across participants, and cue‐behavior repetition was associated with both habit strength and daily step count.

With respect to physical activity identity, two studies have examined changes in identity following new exercise (9 weeks; Wierts, Kroc, & Rhodes, 2024) and running program (10 weeks; Priebe et al., 2020) initiates. For the new exercisers (Wierts, Rhodes, et al., 2024), the initiates showed a small, nonsignificant increase in physical activity identity across 9 weeks, and while MVPA was associated with identity across time, the association was no longer significant when controlling for the random intercept. Among running program initiates, participants showed a significant increase in running identity across the 10‐week program and these increases were associated with increases in running frequency (Priebe et al., 2020). Clearly, this mixed and limited research literature warrants ongoing research to better discern changes in physical activity habit and identity among new MVPA initiates. Furthermore, there is a need to explore these constructs with samples in early parenthood to understand whether physical activity habit and identity can change in this demographic as a result of enrollment in a new physical activity program. Finally, no research has explored physical activity habit and identity formation in recreational sport. Given the psychosocial benefits of sport participation, while considered against the low prevalence of adult involvement in sport, there is a need to understand habit and identity formation and development to better inform their relationship with sport participation across time.

Thus, the purpose of this secondary analysis was to examine the change in physical activity habit and identity among parents of young (<13 years of age) children who participated in a randomized controlled trial that was designed to evaluate the efficacy of three conditions (sport team condition, individual physical activity condition, control condition) for promoting mental health and relationship satisfaction across 3 months. The results of the original pre‐registered research questions showed that team sport participation resulted in improvements in mental health and increased relationship satisfaction compared with the individual physical activity condition and control condition (Rhodes, Beauchamp, et al., 2024). In addition, MVPA significantly increased for those in the individual PA and team sport conditions over time, while the control condition remained stable (Rhodes et al., submitted).

For this study, we first sought to examine the change in physical activity habit and identity over the 3‐month intervention period, test whether changes in habit and identity were predicted by MVPA, and explore whether habit and identity changes interacted with the assigned condition of the participants. We hypothesized that physical activity habit and identity would increase over time from baseline to 3 months, commensurate with the concept of behavioral maintenance associated with these specific constructs (Kwasnicka et al., 2016; Rhodes & Sui, 2021; Spruijt‐Metz et al., 2015). More specifically, we hypothesized that participants assigned to the team sport and individual physical activity conditions would show a significantly steeper change in physical activity habit and identity than the control condition because these conditions comprised initiates of physical activity programming. Finally, because habit and identity are hypothesized to develop, in part, from behavioral experience (Burke, 2006; Wood & Runger, 2016), we hypothesized that MVPA participation would be associated with physical activity identity and habit across the 3‐month study period.

## METHODS

The full detailed methods for this study are reported previously (Grant et al., 2020; Rhodes, Beauchamp, et al., 2024). The study was approved by the University of Victoria Human Research Ethics Board, and the design, conduct, and reporting of the trial followed the Consolidated Standards of Reporting Trials guidelines (Schulz et al., 2010). The main and secondary outcomes of the trial were registered with the Clinical Trials Registry at the National Library of Medicine Identifier: NCT02898285.

### Design

A three‐arm parallel, single‐blinded randomized controlled trial design was conducted. Participants were randomized using a 1:1:1 allocation ratio to one of three groups: (1) team sport condition, (2) individual PA condition, and (3) “date night”/personal time control condition for a duration of 3 months (Grant et al., 2020; Rhodes, Beauchamp, et al., 2024). Parent couples who wanted to participate in the study were randomly assigned at the level of the couple to reduce experimental contamination. The primary and secondary outcomes for the trial corresponded with assessments of differences in mental health and well‐being among the trial conditions, changes in MVPA, and subsequent prediction of MVPA using the Sport Commitment model (Scanlan et al., 1993); these results of which are reported previously (Rhodes, Beauchamp, et al., 2024; Rhodes et al., n.d.). Measures in all three groups were assessed at baseline, and post‐randomization at 6 weeks and 3 months. Recruitment began in November 2016 and was officially put on hold effective April 2020 due to the COVID‐19 pandemic. We were able to resume recruitment in April 2022 up until our end date of April 2023.

### Participants

Participants were recruited mainly through advertisements via online interest sites and social media. Print advertisements were also posted at recreation centers, health care centers, children's recreation classes, and coffee shops regularly throughout greater Victoria, Canada. Pamphlets were also offered at booths at local community markets and family‐oriented events (biweekly). Finally, we engaged in a referral system whereby current participants were invited to pass on study information to others.

#### Inclusion criteria

Participants were eligible if they reported they were a parent of at least one child under 13 years of age who resided in their home. All parenting structures were eligible (i.e., inclusive of those with single‐parent status). Parents needed to report that they were not engaging in regular sport participation (defined as having not participated in an organized sport in the month prior to baseline) and confirm, at the point of screening, that they failed to meet the recommended MVPA guidelines for public health of 150 min of weekly of MVPA (Ross et al., 2020). Parents also needed to be deemed safe to engage in MVPA as assessed via the Get Active Questionnaire (Canadian Society for Exercise Physiology, 2017).

### Measures

#### Outcomes: physical activity habit and identity

Habit was assessed using the four‐item self‐reported automaticity index (Gardner et al., 2012) based on the self‐reported index of habit strength (Verplanken & Orbell, 2003), and identity was assessed using the three‐item role identity subscale (Wilson & Muon, 2008) from the exercise identity scale (Anderson & Cychosz, 1994). Both measures were scored on Likert type scales, adapted to 7‐point scaling, from strongly disagree (1) to strongly agree (7), and behavior was framed as “physical activity/sport participation” inclusive across conditions. Example items of the self‐reported automaticity index are “Physical activity/Sport participation is something that I do automatically” and “Physical activity/Sport participation is something that I do without thinking.” Example items of the role identity subscale are “When I describe myself to others, I usually include my involvement in physical activity/sports” and “Others see me as someone that regularly participates in physical activity/sports.” Reliabilities were acceptable for both habit (time 1 *α* = .94; time 2 *α* = .96, time 3 *α* = .95) and identity (time 1 *α* = .92; time 2 *α* = .93, time 3 *α* = .93).

#### Time‐varying covariate: minutes of sports and MVPA

Minutes of MVPA and sport participation was measured by a modified Godin Leisure‐Time Questionnaire (Godin et al., 1986; Godin & Shephard, 1985). Both weekly frequency and duration of PA and sport participation were provided with an open‐ended assessment, and the multiplicative (frequency × duration) sum of moderate and vigorous intensity minutes, pursued over the previous week, was used as the estimate of weekly MVPA at each of the three timepoints (Courneya et al., 2004). This measure is one of the most widely used self‐report instruments for assessing MVPA because it is easily administered, brief, and has long‐standing concurrent validity evidence based on various criteria including objective activity monitors and fitness indexes (Jacobs et al., 1993).

### Interventions

Detailed information about the interventions can be found in prior publications (Grant et al., 2020; Rhodes, Beauchamp, et al., 2024). In brief, participants who were randomized to the *team sport condition* were asked to make a selection from a customized and up‐to‐date handout of available adult team sport programs in greater Victoria, Canada. These types of activities included but were not limited to basketball, soccer, volleyball, dragon boating, and hockey. Participants randomized to the *individual physical activity condition* were provided with a customized handout of adult individual physical activities and asked to choose from one of these available options. Such individual PAs included but were not limited to rowing, running, resistance training, cycling, swimming, and rock climbing alone. Finally, participants in the “date night” *control condition* were asked to use the honoraria funds to treat themselves to a weekly “night out” or “personal time” of their choice, so long as the time was devoted to be not physically active and the time was spent away from their children. Examples for personal time were provided such as going for coffee, going to the movies, and taking a workshop or class (e.g., cooking class).

### Procedures

As noted on the recruitment information, those interested in taking part in the study were invited to contact the project coordinator via email or phone. Participants were booked for a baseline meeting in the lab of the principal investigator after they were deemed eligible. At this meeting, informed consent was obtained, and participants were asked to complete the baseline assessments. Participants were then randomized into one of the three conditions. Participants were sent follow‐up questionnaire (links to complete via email) after the initial 6 weeks of intervention and followed a similar protocol at the 3‐month trial end point. Incentives for participation included honoraria that were provided to subsidize the cost of the activity (up to a total of $80 CDN) and assistance with childcare costs, up to $25 CDN weekly, if needed.

### Analysis plan

All analyses were conducted using SPSS (28.0). To examine the relationships between MVPA and both physical activity identity and habit, a series of generalized linear mixed models (GLMMs) were conducted (Raudenbush & Bryk, 2002; Zaidman‐Zait & Zumbo, 2013). Participants were included in a given analysis if they had data for at least one of the three assessments at baseline, 6 weeks, and 3 months. As the data are unbalanced, the GLMMs were conducted using maximum likelihood estimation with robust standard errors and the satterthwaite approximation method to compute degrees of freedom (IBM, 2024). Analyses in our previous report (Rhodes et al., submitted) showed that MVPA was significantly positively skewed and kurtotic (zero inflated). Therefore, using Canada's physical activity guidelines (Ross et al., 2020), minutes of MVPA was dichotomized into 0 is <150 min per week and 1 is ≥ 150 min per week.

The first model regressed physical activity identity onto a random intercept, dyad (0 = Do not have a partner in the study; 1 = Have a partner in the study), condition treated as categorical (0 = Date Night; 1 = Individual PA; 2 = Team Sport), MVPA (0 is < 150 min per week of MVPA; 1 is ≥ 150 minutes per week of MVPA) treated as a time‐varying covariate, and a continuous linear trend (0 = baseline; 1 = 6 weeks; 2 = 3 months). This model provided the overall effect of MVPA and condition on identity. The second model added an interaction term between MVPA and the linear trend. This model indicated whether MVPA significantly predicted physical activity identity at baseline, whereas the interaction term indicated whether the strength of the MVPA/identity relationship became significantly stronger, weaker, or remained the same over time. The third model removed the MVPA × linear trend interaction; however, it added a condition × linear trend interaction. Here, the change in identity for participants in the date night condition was compared with the change for participants in the individual PA and team sport conditions. A subsequent GLMM made individual sport the comparison condition in order to make the individual PA versus team sport comparison. Pairwise comparisons were then examined to identify potential condition effects at each time point, whereas follow‐up within‐condition GLMMs were conducted to determine if identity changed from baseline to 6 weeks and 6 weeks to 3 months. Model fit was compared using the Akaike corrected information criterion (AIC_c_), Bayesian information criterion (BIC), and the −2 log likelihood with smaller values representing a better fit (Burnham & Anderson, 2004; Muller et al., 2013). Finally, model assumptions were examined via the residuals for the best fitting model (Quebec Centre for Biodiversity Science, 2023). The same approach then was used to analyze physical activity habit.

## RESULTS

### Participant flow

As previously reported (Rhodes, Beauchamp, et al., 2024), 237 parents contacted the research team about participating in the study, and 204 met the inclusion criteria (of which 114 participants were parent couples). These participants completed baseline measurement and were randomly assigned to one of the three conditions (*n* = 65 in the team sports condition; *n* = 66 in the individual PA condition, and *n* = 73 in the control condition). Of the 204 participants who started the study, 152 participants completed the study to the 3‐month endpoint (75% retention). Of note, due to an internet software migration error at our university systems management, we were unable to retrieve the baseline data of 20 participants, but this error was distributed randomly across all three conditions, so there was no difference in missing data by condition (*p* > .25). No participants cited harms associated with the study.

### Baseline characteristics of respondents

Participants reported a mean age of 37.73 years (SD = 7.12), and 32.4 per cent were male. Most participants were university educated (75%), white (83.6%), employed (66.3%), married or common law (89.1%), and above $75,000 CDN per year income (76.3%). For health behaviors, participants reported an average of 6.82 (SD = 1.07) h of sleep per night, were nonsmokers (96.2%), and self‐reported a mean MVPA of 140.65 (SD = 147) min at baseline. Parents also reported minimal health conditions (<5% reported heart disease, T2 diabetes, cancer, high cholesterol or high blood pressure).

### Predicting physical activity identity

**FIGURE 1:**
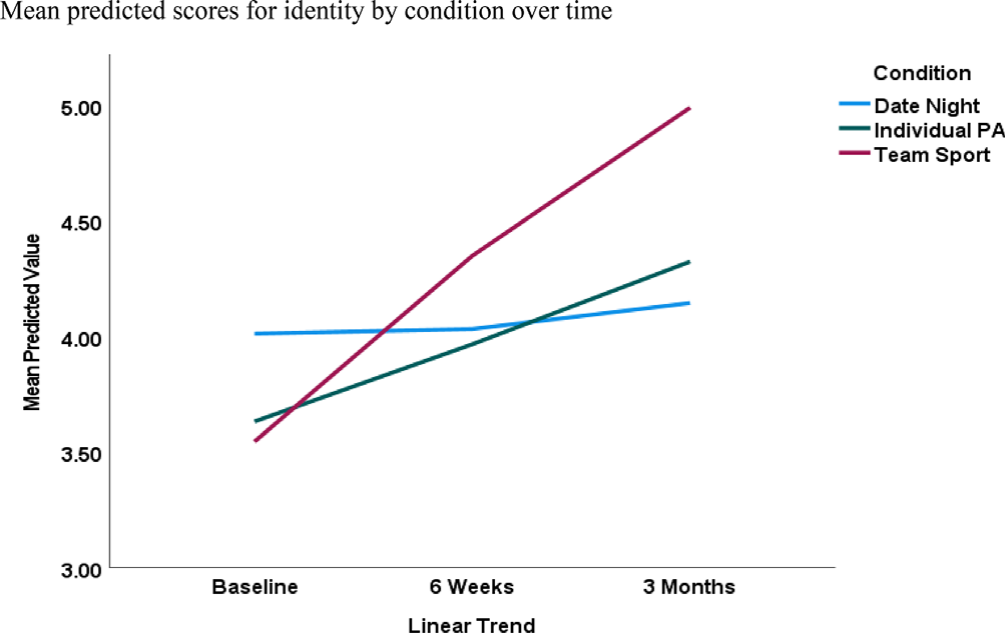
Predicted physical activity identity scores over 3 months comparing team sport, individual physical activity, and “date‐night” control conditions.

As can be seen from Table 1, the first model showed that there were significant main effects for MVPA (beta = .62, *p* < .01) and the linear trend (beta = .23, *p* < .01), whereas model 2 indicated that the strength of the MVPA/identity relationship was stable over time (beta = −.15*, p* = .15). The main effect for MVPA remained significant in model 3 (beta = .50, *p* < .01). There were significant condition × linear trend interactions between the date night and individual physical activity (beta = .35, *p* < .01) and the date night and team sport (beta = .59, *p* < .01) conditions, such that the increase in physical activity identity was greater among participants in the individual physical activity and team sport conditions compared with the date night condition. The subsequent GLMM, which compared the condition (i.e., individual PA versus team sport) × linear trend interaction was nonsignificant (beta = .24, *p* = .06). As can be seen from Figure 1, the means and standard deviations of the predicted scores (Table S1), and the between‐condition pairwise comparisons (Table 3), participants in the team sport condition had significantly higher identity scores (contrast estimate = −.78, *p* = .01) compared with participants in the date night condition at 3 months. However, no other pairwise comparisons were significant. The within‐condition GLMMs (Table S2) showed that identity scores significantly increased from 6 weeks to 3 months (beta = .63, *p* < .01) for participants in the individual PA condition, whereas physical activity identity scores significantly increased from baseline to 6 weeks (beta = .73, *p* < .01) and 6 weeks to 3 months (beta = .44, *p* = .02) for participants in the team sport condition. Finally, the residuals indicated that model assumptions were met.

**TABLE 1:** Results from the final generalized linear mixed models predicting identity.

Parameter	Model 1	Model 2	Model 3
	Beta (95% CI)	Beta (95% CI)	Beta (95% CI)
Intercept	3.43 (2.95 to .91)**	3.36 (2.89 to 3.84)**	3.72 (3.23 to 4.20)**
Dyad			
Do not have a partner in study	0	0	0
Have a partner in study	.17 (−.27 to .61)	.17 (−.27 to .61)	.19 (−.25 to .63)
MVPA			
< 150 min/week	0	0	0
≥ 150 min/week	.62 (.38 to .86)**	.76 (.45 to 1.07)**	.50 (.27 to .73)**
Condition			
Date night	0	0	0
Individual PA	−.13 (−.67 to .41)	−.12 (−.66 to .42)	−.44 (−1.04 to .15)
Team sport	.09 (−.44 to .63)	.11 (−.43 to .64)	−.39 (−.98 to .20)
Linear trend	.23 (.13 to .34)**	.31 (.15 to .47)**	−.03 (−.18 to .12)
Linear trend * MVPA	‐	−.15 (−.37 to .06)	‐
Condition * linear trend			
Date night × linear trend	‐	‐	0
Individual PA × linear trend	‐	‐	.35 (.14 to .56)**
Team sport × linear trend	‐	‐	.59 (.34 to .83)**
Fit index			
AIC_c_	1646.40	1646.99	1626.26
BIC	1654.74	1655.32	1634.58
−2 log likelihood	1642.38	1642.96	1622.23

Abbreviations: AIC_c_, Akaike corrected information criterion; BIC, Bayesian information criterion; CI, confidence interval; MVPA, moderate to vigorous physical activity; PA, physical activity.

** *p* < .01.

### Predicting physical activity habit

**FIGURE 2:**
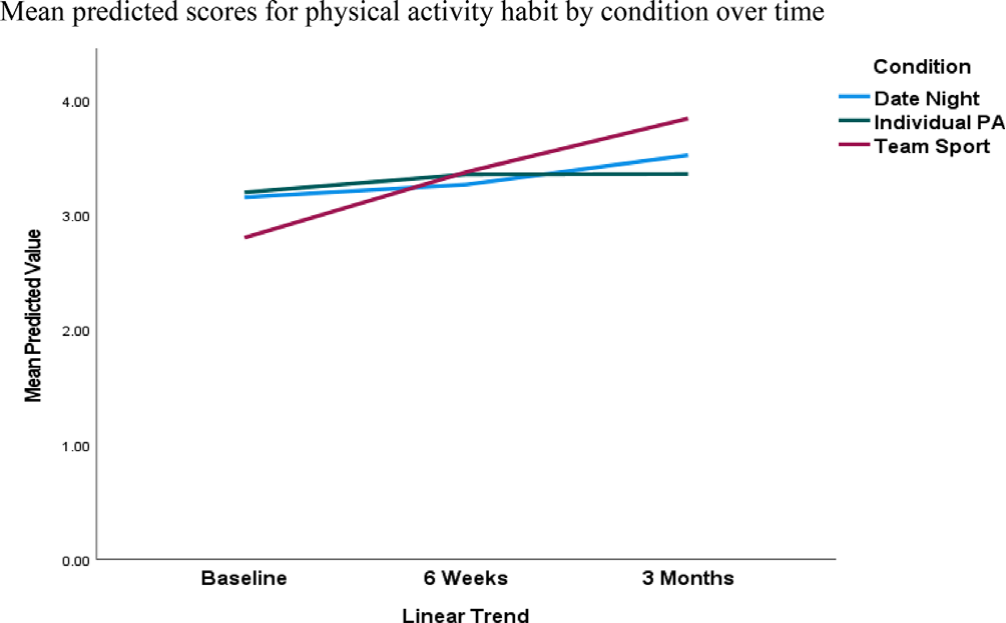
Predicted physical activity habit scores over 3 months comparing team sport, individual physical activity, and “date‐night” control conditions.

Similar to physical activity identity, the first model (see Table 2) showed that there was a significant main effect for MVPA (beta = .66, *p* < .01) and the linear trend (beta = .18, *p* < .01), whereas the second model showed that the strength of the MVPA/habit relationship was stable over time (beta = −.10, *p* = .37). The main effect for MVPA remained significant in model 3 (beta = .62, *p* < .01). There were also significant condition × linear trend interactions when comparing the date night versus team sport conditions (beta = .34, *p* = .02) and the individual physical activity versus team sport conditions (beta = .42, *p* = .01), such that habit increased to a greater extent for those in the team sport versus date night and individual physical activity conditions (see Table S3 for the means and standard deviations of the predicted scores and Figure 2). The between‐condition pairwise comparisons were nonsignificant (see Table 3); however, the within‐condition GLMMs (Table S4) showed that the physical activity habit score significantly increased from 6 weeks to 3 months (beta = .48, *p* = .02) for participants in the team sport condition. Residual plots indicated that model assumptions were met.

**TABLE 2:** Results from the final generalized linear mixed models predicting habit.

Parameter	Model 1	Model 2	Model 3
	Beta (95% CI)	Beta (95% CI)	Beta (95% CI)
Intercept	2.77 (2.35 to 3.19)**	2.72 (2.30 to 3.15)**	2.84 (2.42 to 3.27)**
Dyad			
Do not have a partner in study	0	0	0
Have a partner in study	.10 (−.29 to .49)	.10 (−.29 to .49)	.10 (−.30 to .49)
MVPA			
<150 min/week	0	0	0
≥150 min/week	.66 (.40 to .93)**	.76 (.41 to 1.10)**	.62 (.37 to .87)**
Condition			
Date night	0	0	0
Individual PA	−.05 (−.53 to .43)	−.05 (−.52 to .43)	.02 (−.51 to .55)
Team sport	−.01 (−.49 to .47)	−.003 (−.48 to .48)	−.28 (−.80 to .25)
Linear trend	.18 (.07 to .29)**	.23 (.08 to .38)**	.12 (−.04 to .28)
Linear trend * MVPA	‐	−.10 (−.32 to .12)	‐
Condition * linear trend			
Date night × linear trend	‐	‐	0
Individual PA × linear trend	‐	‐	−.08 (−.31 to .16)
Team sport × linear trend	‐	‐	.34 (.06 to .62)*
Fit index			
AIC_c_	1659.43	1661.17	1653.90
BIC	1667.76	1669.50	1662.22
−2 log likelihood	1655.40	1657.14	1649.87

Abbreviations: AIC_c_, Akaike corrected information criterion; BIC, Bayesian information criterion; CI, confidence interval; MVPA, moderate to vigorous physical activity; PA, physical activity.

*Note*:

* *p* < .05.

** *p* < .01.

**TABLE 3:** Pairwise comparisons for condition at each time point for physical activity identity and habit.

Contrast	Baseline	6 weeks	3 months
	Estimate (95% CI)	Estimate (95% CI)	Estimate (95% CI)
Habit			
Date night–individual PA	.44 (−.15, 1.04)	.09 (−.46, .64)	−.25 (−.84, .33)
Date night–team sport	.39 (−.20, .98)	−.19 (−.73, .34)	−.78 (−1.37, −.19)**
Individual PA–team sport	−.05 (−.64, .54)	−.29 (−.80, .23)	−.52 (−1.07, .03)*
Identity			
Date night–individual PA	−.02 (−.55, .51)	.06 (−.42, .54)	.14 (−.41, .68)
Date night–team sport	.28 (−.25, .80)	−.06 (−.55, .43)	−.40 (−1.01, .20)
Individual PA–team sport	.30 (−.24, .83)	−.12 (−.60, .36)	−.54 (−1.11, .05)

*Note*: The least significant difference adjusted *p*‐level was used.

Abbreviations: %, per cent; CI, confidence interval; PA, physical activity.

*Note*:

* *p* = .06.

** *p* < .01.

## DISCUSSION

The purpose of this exploratory analysis was to examine the change in physical activity habit and identity among parents of children (< 13 yrs) who participated in team sport, individual physical activity, or date‐night control conditions as part of a preregistered randomized trial across 3 months. An understanding of physical activity habit and identity changes over time is important to inform factors that may be instrumental in maintaining the behavior after the initial start of participation (Dunton et al., 2022; Rhodes & Sui, 2021). This is also the first study, to our knowledge, to explore physical activity habit and identity changes in recreational sport participation and one of only a handful of prior studies to track these constructs over time among new MVPA initiates (Baretta et al., 2024; Kaushal & Rhodes, 2015; Priebe et al., 2020; Wierts, Kroc, & Rhodes, 2024). Understanding purported maintenance constructs, such as identity and habit (Kwasnicka et al., 2016), may also be highly relevant to parents of young children because physical activity declines during early parenthood (Carson et al., 2018), and thus, any new initiative to promote physical activity will likely need to understand continuation of the behavior for ongoing effectiveness.

We first sought to examine the changes in physical activity habit and identity over this 3‐month period. Commensurate with the concept of behavioral maintenance associated with these specific constructs (Kwasnicka et al., 2016; Rhodes & Sui, 2021; Spruijt‐Metz et al., 2015), we hypothesized that habit and identity would increase over time from baseline to 3 months. However, we also sought to explore whether physical activity habit and identity changes interacted with the assigned conditions of the participants. We hypothesized that participants assigned to the team sport and individual physical activity conditions would show a significantly steeper change in habit and identity than the control condition because these conditions comprised initiates of physical activity programming. This hypothesis was partly supported. Specifically, condition interacted with the linear trend for physical activity identity, such that individuals in the team sport and individual physical activity conditions demonstrated greater increases in identity compared with individuals in the date night control condition. Moreover, between‐condition pairwise comparisons showed that participants in the team sport condition had significantly higher physical activity identity scores compared with participants in the date night condition at 3 months. For team sports, this differential change in identity increased from baseline to 6 weeks and then again from 6 weeks to 3 months. For participants assigned to the individual physical activity participation condition, identity scores significantly increased from 6 weeks to 3 months. For physical activity habit, there was also a significant condition by linear trend interaction, such that participants in the team sport condition demonstrated a greater increase in habit, compared with participants in the date night and individual PA conditions, over the 3‐month study period. However, between‐condition pairwise comparisons were nonsignificant at each time point. Within‐condition analyses showed that habit significantly increased from 6 weeks to 3 months for participants in the team sport condition only. Thus, the effects of condition on habit were considerably more attenuated in comparison to identity and only marked within the team sport condition.

The findings add to an emerging literature showing that physical activity habit (Kaushal & Rhodes, 2015) and identity (Priebe et al., 2020) can change among new physical activity initiates, but change is sometimes inconsistent (Wierts, Kroc, & Rhodes, 2024). From a theoretical perspective, habit and identity are generally considered resistant to change (Burke, 2006; Verplanken et al., 1998), partly supporting why these constructs are often linked to the concept of maintenance (Verplanken & Sui, 2019). However, evidence that these constructs can be modified over time in new initiates of a MVPA program, particularly team sports, is promising.

This is also useful information when considering the sample of parents with young children who took part in this study. The lifestyle demands of parenthood are generally considered the overwhelming cause for dropping physical activity participation (Bellows‐Riecken & Rhodes, 2008; Candelaria et al., 2012). Parenting young children is often considered a chaotic time in adults' lives with constant attention to ongoing stressors (Parfitt & Ayers, 2014; Pollman‐Schult, 2014). From a theoretical perspective, this instability in life routine is not particularly conducive to physical activity habit formation because cues (internal and external) may shift more often for parents compared with populations with a more predictable schedule (Rhodes, 2024; Wood et al., 2005). This may explain, in part, why the change for habit was attenuated. Parenting, however, is also often accompanied by the development of a strong identity (I am now a mother, I am now a father) that supplants other identities in one's self‐concept due to the typical reallocation of one's time and/or priorities (McMahon, 1995). It is promising evidence that physical activity identity can show significant change among parents under these conditions over 3 months of physical activity participation after enrolment in a new program.

When interpreting the differential effects of physical activity habit and identity from the sport versus individual physical activity conditions, we caution that the study was not designed to specifically intervene upon identity or habit, so any comparisons of the changes between the two constructs, by condition, is conjecture. The more robust findings for physical activity identity and team sports, however, may be due to the nature of this activity. Specifically, one of the major differences between the team sport condition and the individual physical activity condition is the social properties of team sports. Social aspects of physical activity have been linked to physical activity self‐identity in past research (Evans et al., 2019; Rhodes et al., 2016; Wierts, Rhodes, et al., 2024) and this may also be linked to a larger, developing social physical activity identity in the team sports condition (Beauchamp, 2019; Beauchamp & Rhodes, 2020). In addition, team sports have a specific rule‐set (Eime et al., 2010), allowing for self‐classification (e.g., I am playing hockey, I am playing soccer), which has been linked to identity formation more than individual physical activity (e.g., I am walking more each week, I am jogging more) (West & Brown, 2013). Finally, both physical activity conditions, in the context of early parenthood, represent a challenge to enact (Nomaguchi & Milkie, 2003). A behavioral identity is theorized to strengthen under conditions of success under challenge (Caldwell et al., 2018; Kendzierski & Morganstein, 2009; Rhodes et al., 2023), while habit is conceived to strengthen more under circumstances of stability (Rebar et al., 2023). Certain prerequisite aspects of habit formation were present in the physical activity conditions (e.g., behavioral performance at least once per week, same activity across 3 months likely provided a consistent cue schedule), likely attributing to why habit showed some increase, but the characteristics of team sport within the backdrop of the challenges of parenthood may explain why identity had a larger and more pronounced change than habit. Future research focused on the properties and underlying behavior change techniques of physical activity habit formation (see Ma et al., 2023 for a recent review), and identity formation (see Rhodes, Wierts, et al., 2024 for a recent review) is needed to specifically explore optimization.

Our additional research question was to test whether physical activity habit and identity were predicted by MVPA across the trial. Because habit and identity are hypothesized consequences, in part, of behavior (Burke, 2006; Wood & Runger, 2016), we hypothesized that MVPA participation would predict habit and identity across time. This hypothesis was supported, and the relationship between behavior and identity/habit was stable, even after controlling for the main effect of condition. Theoretically, this supports the conceptualization of these constructs as experiential, where behavior itself is an important ingredient to identity and habit (Bem, 1972; Wood et al., 2002). Practically, however, the challenge for health promoters will be to facilitate conditions during physical activity that optimize the development of habits (Lally & Gardner, 2011) and identity (Caldwell et al., 2018). For example, habit formation in physical activity is likely a consequence of performance under consistent conditions, with a salient cue structure, and relatively low behavioral complexity (Kaushal & Rhodes, 2015; Rebar et al., 2023; Rhodes & Rebar, 2018). Behavioral identity formation has seen less theoretical attention than habit, but current evidence suggests identity is a result of perceived capability, investment, self‐regulation, and social conditions that act as behavioral comparators, and alignment with other values (Strachan et al., n.d.). Sustained research exploring these potential determinants of identity and habit, across different populations and alongside behavioral performance, is warranted.

Despite the strengths of this exploratory analysis, there are noteworthy limitations. First, while a strength of our study was its pragmatism in accepting parents of all family systems and situations (i.e., directly relevant to what a recreation program is likely to encounter with its registrants), the partially clustered data (some participants had partners, others did not) posed an analytical challenge. Our analyses used a dummy coded dyad variable as a covariate to “partially” capture this variance, yet we recommend a future study designed specifically to explore different parent family structures on habit and identity outcomes to better understand these potential effects. Second, physical activity was measured with self‐report and may be subject to responding biases (Prince et al., 2008). Our PA measure was skewed and had to be dichotomized for use in our models (Ross et al., 2020). Some of this skew may be a result of the trial design. Specifically, most of our participants were inactive at baseline (as per inclusion criteria) which manifested in zero inflation. Additional research with direct measures of PA may be prudent to replicate these effects, and future research applying a continuous MVPA measure would improve our understanding of dose–response relationships between MVPA and habit/identity changes. Finally, our sample was moderately active, mainly white, middle income, and university educated. While many of these features do represent Greater Victoria (Statistics Canada, 2017), the generalizability of these results to more diverse socioeconomic conditions of parents are unknown.

In summary, physical activity identity, and to a lesser extent, habit, showed an increase across 3 months among parents with children (<13 years of age), who were participating in a randomized trial. These changes, however, interacted with group condition assignment. Physical activity identity increased for participants in the team sport condition across the 3 months and was significantly different (at 3 months) from a stable identity profile among those assigned to the control condition. Participants in the individual physical activity condition increased identity from 6 weeks to 3 months. By contrast, physical activity habit had a more attenuated change across time by condition, with participants in the team sport condition showing increased habit from 6 weeks to 3 months. Across all conditions, past MVPA predicted identity and habit changes. The findings contribute to our understanding of theoretical maintenance processes, adding to a small number of studies on physical activity habit and identity changes among physical activity program initiates and representing the first exploration of team sport participation on these constructs. Interventions that can help support parents to participate in team sport may be particularly effective to foster physical activity identity and to a lesser extent, habit, in this population.

## CONFLICT OF INTEREST STATEMENT

The authors declare no conflicts of interest.

## ETHICS STATEMENT

This study was approved by the IRB from the University of Victoria, Canada. All participants provided ongoing informed consent.

## DATA AVAILABILITY STATEMENT

Data is available upon reasonable request from the primary author. Full shared access to these data is not available to due limitations of the original participant consent.

## Supporting information


**Table S1.** Means and standard deviations of the predicted scores for Identity from model 3 in Table 1.
**Table S2.**Follow‐up generalized linear mixed models examining the change in Identity within condition.
**Table S3.** Means and standard deviations of the predicted scores for Habit from model 3 in Table 2.
**Table S4.** Follow‐up generalized linear mixed models examining the change in Habit within condition.
