# Supplementary material for: Changes in identity and habit formation during 3 months of sport and physical activity participation among parents with young children
Source: Appl Psychol Health Well Being. 2025 Feb 11;17(1):e70009. doi: 10.1111/aphw.70009 (PMC11813645; doi:10.1111/aphw.70009)
Supplement: Supplementary file 1 — Table S1. Means and standard deviations of the predicted scores for Identity from model 3 in Table 1. Table S2.Follow‐up generalized linear mixed models examining the change in Identity within condition. Table S3. Means and standard deviations of the predicted scores for Habit from model 3 in Table 2. Table S4. Follow‐up generalized linear mixed models examining the change in Habit within condition. [file APHW-17-0-s001.docx]

**====================================================================**

**IDENTITY**

**====================================================================**

**Supplemental Table 1**

Means and standard deviations of the predicted scores for Identity from model 3 in Table 1

|  | Baseline | 6 weeks | 3 months |
| --- | --- | --- | --- |
| Condition | Mean ± SD | Mean ± SD | Mean ± SD |
| Date Night | 4.02 ± 1.70 | 4.03 ± 1.74 | 4.15 ± 1.71 |
| Individual Sport | 3.63 ± 1.50 | 3.97 ± 1.56 | 4.32 ± 1.49 |
| Team Sport | 3.55 ± 1.36 | 4.35 ± 1.38 | 4.99 ± 1.31 |

Note. SD = standard deviation.

**Supplemental Table 2**

Follow-up generalized linear mixed models examining the change in Identity within condition

|  | Date Night | Individual PA | Team Sport |
| --- | --- | --- | --- |
| Contrast | Beta^1^ (95% CI) | Beta^1^ (95% CI) | Beta^1^ (95% CI) |
| Baseline to 6 weeks | .06 (-.24, .36) | .07 (-.26, .40) | **.73 (.29, 1.18)**** |
| 6 weeks to 3 months | -.09 (-.37, .18) | **.63 (.23, 1.02)**** | **.44 (.10, .77)*** |

Note. * p < .05; **p<.01; % = percent; CI = confidence interval; PA = physical activity.

^1^ Betas are adjusted for dyad and meeting the moderate to vigorous PA guideline (or not).

**====================================================================**

**HABIT**

**====================================================================**

**Supplemental Table 3**

Means and standard deviations of the predicted scores for Habit from model 3 in Table 2

|  | Baseline | 6 weeks | 3 months |
| --- | --- | --- | --- |
| Condition | Mean ± SD | Mean ± SD | Mean ± SD |
| Date Night | 3.15 ± 1.42 | 3.26 ± 1.46 | 3.52 ± 1.40 |
| Individual Sport | 3.19 ± 1.29 | 3.34 ± 1.31 | 3.35 ± 1.32 |
| Team Sport | 2.80 ± 1.24 | 3.37 ± 1.23 | 3.83 ± 1.20 |

Note. SD = standard deviation.

**Supplemental Table 4**

Follow-up generalized linear mixed models examining the change in Habit within condition

|  | Date Night | Individual PA | Team Sport |
| --- | --- | --- | --- |
| Contrast | Beta^1^ (95% CI) | Beta^1^ (95% CI) | Beta^1^ (95% CI) |
| Baseline to 6 weeks | .11 (-.27, .49) | -.21 (-.55, .13) | .36 (-.20, .92) |
| 6 weeks to 3 months | .07 (-.23, .37) | .25 (-.10, .60) | **.48 (.07, .90)*** |

Note. * p < .05; % = percent; CI = confidence interval; PA = physical activity.

^1^ Betas are adjusted for dyad and meeting the moderate to vigorous PA guideline (or not).
